# Ketamine assisted psychotherapy in postpartum mood and anxiety disorders: a limited case series

**DOI:** 10.3389/fpsyt.2025.1661604

**Published:** 2026-01-22

**Authors:** Alka Christnacht, Therry Rose Eparwa, Emily Whinkin, Sunil Aggarwal

**Affiliations:** 1 College of Nursing, Seattle University, Seattle, WA, United States; 2 Advanced Integrative Medical Science Institute (AIMS) Institute, Seattle, WA, United States; 3 University of Washington, Department of Geography, Seattle, WA, United States

**Keywords:** ketamine, ketamine assisted psychotherapy, KAP, postpartum, peripartum, anxiety, depression, PMAD

## Abstract

The postpartum period is notorious for rapid and profound changes for birthing individuals and their families. Significant shifts to hormonal and physical health, routines and family roles, and the salience of personal and community risk factors all contribute to potential psychiatric and psychological distress for parents, sometimes diagnosed as a postpartum mood or anxiety disorder (PMAD). Existing pharmacologic treatment modalities for PMADs do not comprehensively address the profound shifts of the postpartum period, often inadequate at reaching peak therapeutic efficacy in a shorter time frame, in patient accessibility, or offering sustained benefit. Ketamine assisted psychotherapy (KAP), is increasingly recognized as a therapeutic option for a range of psychiatric disorders and has been explored as a modality to *prevent* PMADs when used during Cesarean births. This retrospective case series examines three cases where ketamine assisted psychotherapy (KAP) was introduced during the first two years postpartum as part of a comprehensive treatment plan for PMADs. This study describes the utility of ketamine assisted psychotherapy to the postpartum individual, and offers perspective on the impact of psychedelic insights through treatment of PMAD symptoms

## Introduction

Postpartum mood and anxiety disorders (PMADs) is a term used to describe mental illnesses that occur around the postpartum period. The criteria for PMADs vary. The Diagnostic and Statistical Manual of Mental Disorders, 5th edition text-revision (DSM-5-TR; 2) simply adds the specifier ‘with peripartum onset’ to encapsulate a mood disorder that begins either in pregnancy or postpartum. However, many prominent organizations define PMADs as starting within the first several weeks following childbirth and occurring up to a year following childbirth (2–4). PMADs affect nearly 1 in 5 (5), often being accompanied by profound physical and life changes for the birthing individual as well as the child and family system. PMADs may increase the risk of adverse physiological and psychological health outcomes such as suicide, a leading cause of maternal death in the perinatal period within the United States (6, 7). PMADs are associated with lower initiation rates for breastfeeding, which can lead to poorer maternal-infant bonding (8). Effects of PMADs on children include increased morbidity and mortality rate; delays in cognitive, motor, emotional, and language development (8); and delays in communication and personal-social development (9). PMADs can have a reciprocal relationship with paternal mental health. The risk of depression nearly doubles in the postpartum period for fathers, with a quarter to half of fathers experiencing paternal depression if their partner is experiencing a PMAD (10), and poor paternal mental health can often be a risk factor for PMADs (11).

Social and psychological risk factors for PMADs include a history of prenatal mood and/or anxiety disorders, high stress or trauma (exposure to life-threatening events), lack of social support, lack of spousal/partner support, immigration or lack of acculturation, and low socioeconomic status (SES). Biological and genetic risk factors include gestational diabetes, increased inflammatory mediators, abnormal neuroendocrine hormone function, and epigenetic changes. Birthing experiences themselves may also precipitate PMADs as negative birth experiences, multiple births, and preterm deliveries have been associated with PMADs (12, 13).

The pathophysiology of PMADs may differ compared to mood and/or anxiety disorders during other periods of life. These emerging findings must be approached with some temperance, as many are derived from single studies. Stress and dysregulation of the hypothalamic-pituitary-adrenal (HPA) axis can play significant roles in triggering depression both in and out of the peripartum period (14). Neurological changes observed in both postpartum depression (PPD) and major depressive disorder (MDD) include decreased activation of reward circuitry, reduced binding of serotonin receptors, and decreased activity of gamma-aminobutyric acid (GABA) (14). In a mice model where PMADs was replicated by maternal separation and early weaning, reduced vesicular transport of GABA and glutamate resulted in increased despair- and anhedonia-like behaviors and disrupted maternal care (15). Specific to PPD is that the amygdala, commonly known as the fear center of the brain, has a blunted response to negative stimuli (16, 17), whereas in MDD the response is typically heightened (18).

Hormonal changes during pregnancy and childbirth may lead to epigenetic changes that increase the risk for PMADs (19). Estrogen-dependent changes are associated with PMADs (14, 20), with lower levels of estradiol correlated with depression and anxiety. Homozygous short alleles for the serotonin transporter gene (SERT) are linked to larger drops in estradiol following childbirth, raising the risk of postpartum depression at 6 weeks (21). Although estradiol naturally declines following childbirth, greater precipitous declines are associated with decreased synaptic plasticity and brain-derived neurotrophic factor (BDNF) expression (22). Circulating levels of the neurosteroid allopregnanolone increase dramatically during pregnancy and drop precipitously following childbirth (23). Allopregnanolone positively modulates GABA-alpha (GABA-A) receptors in the brain (24), producing anxiolytic and antidepressant effects. Individuals with PMADs show low serum levels of allopregnanolone in late pregnancy (15) and it is thought that the upregulation of GABA-A receptors following childbirth does not occur (25).

Current treatment options for PMADs are often generalized from standard depression protocols (e.g., selective serotonin reuptake inhibitors (SSRIs); serotonin-norepinephrine reuptake inhibitors (SNRIs)) and can take several weeks to achieve full efficacy (26). Further, treatments provided during this period must take into account that any drug given to individuals who are lactating must also demonstrate a level of safety for infants (27). Unfortunately, there are limited approved PMAD-specific pharmacologic treatment options that alter neuroactive steroid and hormone levels (28). Thus far, synthetic allopregnanolone-based treatment (e.g., brexanolone and zuranolone) addresses the neuroactive steroid fluctuation seen in PMADs and has demonstrated positive results (29, 30). However, they currently remain inaccessible for many due to time and/or financial barriers. Additionally, manufacturer recommendations advise suspending breastfeeding during treatment, which impacts maternal-infant bonding. In its early phases of study for PMADs, estradiol treatment has demonstrated safety in lactation; however, its efficacy is unclear, it may decrease milk supply, and carry the risk of thromboembolic events (31). Given the potentially serious implications that PMADs can have on families it is crucial to identify alternate pharmacologic treatments that can be utilized for this unique subset of psychiatric disorders. Ketamine has shown a range of therapeutic benefits in various psychiatric and mental health disorders (32).

A dissociative anesthetic, ketamine is listed as an essential medicine by the World Health Organization (33). It is highly lipid-soluble with a low protein-binding affinity (20-50%) (34), allowing it to readily cross the blood-brain barrier. This leads to a rapid onset of action and a short half-life of 1–3 hours (35). Such swift elimination of ketamine from the system reduces the duration of drug side effects as well as excretion of the drug in breast milk. Although unrelated to the rapid pharmacokinetics, ketamine additionally is known to provide therapeutic relief for mental health symptoms faster than traditional antidepressants which may take 4–6 weeks for full efficacy (36). The rapid elimination from breast milk is particularly beneficial in individuals with PMADs, facilitating a quicker return to ability to lactate, and support parent-child bonding during a critical period for neurobiological regulation and attachment development (37). In order of decreasing bioavailability, ketamine can be administered intravenously (IV), intramuscularly (IM), intranasally (IN), sublingually (SL), or orally (PO) (34, 38). Its metabolism primarily occurs in the liver, and metabolites are excreted in the urine (34).

The exploration of ketamine’s psychiatric potential began in the 1970s. Landmark studies, such as Khorramzadeh and Lotfy (39), demonstrated that ketamine injections led to remission of various psychiatric symptoms, lasting up to a year, in approximately 100 participants with diverse psychiatric diagnoses. Since then, promising results have emerged from studies investigating ketamine for a range of psychiatric disorders. While ketamine is recognized as a non-competitive antagonist of postsynaptic glutamatergic N-methyl-D-aspartate receptors (NMDAR) (40), the exact mechanisms behind its rapid and sustained antidepressant effects remain unclear. This is evidenced by the inconsistent antidepressant effects observed with other NMDAR antagonists, such as memantine (41, 42). Ketamine’s immediate effects disrupt downstream cellular processes, including gene expression and protein regulation (43). It is proposed to block tonic GABAergic inhibition, increasing glutamate release and cycling, which heightens glutamate transmission into alpha-amino-3-hydroxy-5-methyl-4-isoxazolepropionic acid receptors (AMPARs), activating BDNF protein synthesis and synaptogenesis (44). Lower BDNF levels are correlated with depression, while higher levels promote neuroplasticity (45). Ketamine may exert anxiolytic and antidepressant effects in PMADs (46) by respectively enhancing infralimbic cortex and hippocampus synaptic plasticity and synaptogenesis (15, 33, 47). Ketamine might outperform allopregnanolone synthetics in treating PMADs since synthetic allopregnanolone only reduced behaviors of despair, but ketamine also reversed anhedonia (15), though further studies are needed.

Ketamine potentially has utility as a prophylactic, particularly for PPD, given its frequent anesthetic administration for surgical procedures and rapid, effective antidepressant qualities. Administered at 0.2-0.5 mg/kg IV during or after caesarean sections, sub-anesthetic doses of ketamine generally reduced depression rates 48 hours to 6 weeks postpartum (37), with higher doses leading to even lower depressive symptoms. By contrast, another study found that doses ≥ 0.5 mg/kg of IV ketamine reduced the scores and prevalence of PPD symptoms at 1 week postpartum; however, it was ineffective at maintaining antidepressive effects at 4 weeks postpartum (48). These varied outcomes may be influenced by an individual’s risk probability for a PMAD. Compared to placebo, high-risk birthing individuals who received IV ketamine (0.5 mg/kg) 10 minutes post-delivery had significantly reduced incidences of PPD (13). Moreover, there were no significant differences observed within the low-risk group, and a few individuals still developed PPD.

Ketamine is generally considered a safe medication with a favorable tolerability profile. Its side effects are typically acute and mild, including nausea, vomiting, dizziness, headache, sedation, transient hypertension, blurred vision, agitation, anxiety, and dissociative symptoms (47, 49). Early studies also indicate safety during lactation, where Wolfson et al. (50) found that the relative infant dose (RID) of ketamine, after IM administration of 0.5 mg/kg and 0.1 mg/kg to women, never exceeded 1% when serum metabolites were measured every three hours for up to 12 hours. RID, which measures the proportion of mg/kg/day of the drug received by the infant relative to the lactating individual’s dose, is considered safe at levels up to 10% (51). Given ketamine’s poor oral bioavailability in adults, it was hypothesized that even less would transfer to infants via lactation (50). Regardless, due to limited data, close monitoring of infants consuming milk from lactating individuals receiving ketamine is recommended (52).

Beyond pharmacologic and physiologic effects, additional benefits may arise from ketamine’s experiential effects. Sub-anesthetic doses of ketamine have shown the ability to induce “mystical-type” experiences. These experiences are comparable to spontaneous or “spiritual” phenomena, often characterized by ineffability, metaphysical insight, positive affect, and a heightened sense of connection with the world and others (53–55). Additionally, ketamine’s mood-elevating and antidepressant benefits are prolonged when combined with subsequent psychotherapy, as occurs in ketamine-assisted psychotherapy (KAP) (30, 47, 56).

Despite ketamine’s emergence as a potential treatment option and prophylaxis for certain mood disorders, there is limited literature on its clinical use and coverage for individuals with PMADs. This scarcity may be attributed to a lack of funding or hesitancy to study medication during the perinatal period. Because there are multiple causative agents and circumstances that increase susceptibility to PMADs, each case is unique. Treatment for PMADS must address the distinct physical and psychological concerns of each individual. Multiple mutually-reinforcing causes of PMADs also confers multiple inroads for possible treatments, which are most appropriately utilized as an integrated protocol of best practices across disciplines. It is within this framework of individualized and integrative care that the patients included in this series received adjunctive ketamine assisted psychotherapy for their PMADs symptoms.

## Methods

This study aims to examine a limited number of existing cases of ketamine use in PMADs to gather information regarding its potential benefits, safety, and tolerability as a psychiatric and psychological intervention for this population and the family systems of which they are a part.

### Setting and interventions

The Advanced Integrative Medical Science Institute (henceforth, AIMS) in Seattle, WA, offers private, holistic ketamine therapy for mood disorders and other indications. Most patients seen at this clinic are from the greater Seattle metropolitan area. However, there are some out-of-state patients due to the available hotel partnerships and telehealth appointments for screening, preparation, and integration.

A medical provider performs a thorough medical and psychiatric history and evaluation of the patient prior to ketamine treatment. Contraindications include ketamine allergy, severe renal/hepatic illness, high cardiac risk requiring cardiac monitoring, psychosis history, poorly controlled bipolar disorder, or ketamine/illicit substance use disorder, though exceptions exist for alcoholism, gambling/sex, and nicotine addictions with KAP. Poorly processed PTSD may also be a contraindication.

In-clinic KAP protocols require 4-hour fasting, restroom use before the session, and arranged transport home. Vital signs are taken by an RN prior to the session, but no further cardiac monitoring occurs. Prophylactic anti-nausea medication is optional. The patient sits in a recliner and can choose to have relaxing music and wear an eye mask. A starting ketamine dose is typically 1.5 mg/kg IM or 300 mg SL. In consideration of patient tolerance, medical history, medication interactions, medical indication, or previous psychedelic experience. Doses are increased approximately 0.1-0.2 mg/kg IM or 50–100 mg SL per session until full therapeutic benefit is attained. A licensed mental health clinician observes for 2–3 hours during the dissociative “experiential session,” transcribing actions. At-home KAP uses 300 mg SL ketamine troches, where the patient is encouraged to mimic clinic protocols. In lactating patients, patients are recommended to pump and discard breastmilk for 12 hours post-administration. Within a week of KAP treatment, the patient participates in psychotherapy “integration sessions” to review in-clinic notes and process the experience.

### Participants

The AIMS Medical Outcomes Study (AMOS, NCT04512755; 57) is an observational prospective outcomes study overseen by the Seattle University Institutional Review Board (IRB). Participants for this study were selected using a convenience sampling with the following inclusion criteria: consented to participate in the AMOS study; were seen at AIMS between November 2019 and December 2023; self-reported struggles with a postpartum-related mood and/or anxiety disorder; received a diagnosis of a mood or anxiety disorder; and prescribed ketamine. Any individuals who did not meet all of these criteria were excluded. One individual who did not consent to AMOS but instead gave verbal consent, documented in her medical record, for her outcomes to be used as part of research on peripartum health met all other clinical criteria and was included in the study.

The postpartum period was not restricted to 12 months following childbirth, which is the frequently-described postpartum timeframe. Extant literature indicates that postpartum depressive symptoms can persist and remain elevated for as long as 3 years (57). As the study was anticipated to have a limited sample size, the postpartum timeframe was extended up to 24 months to gather all potentially available data.

Participants were identified via AIMS’ electronic medical record (EMR) system. The EMR analytics enabled a refined search of potential subjects through using specific search terms and diagnostic International Classification of Diseases, 10th revision (ICD-10) codes relevant to PMADs. Individuals were flagged if any of the following diagnoses were explicitly included within their health record: MDD, single episode, unspecified postpartum depression (F32.9); postpartum mood disturbance (O90.6); or postpartum depression (F53.0). The ‘diagnosis contains’ search feature flagged individuals whose diagnoses contained the term “postpartum” or “pregnancy.” Provider recall was also solicited by internal email communications and during a morning huddle to further identify any individuals who may have PMADs.

### Data collection and analysis

Participant information was collected from the EMR and stored on a spreadsheet database, which included: participant identifier, age, marital status, date of delivery, psychiatric diagnoses, medications, and notable medical and/or obstetric history. Any ketamine-related clinical encounter documented by medical providers and licensed mental health clinicians was reviewed to gather appointment dates, narratives detailing PMAD symptoms, response to ketamine treatment, and any effects. The number of appointments and length of treatment for participants significantly varied, depending on whether or not they continued to identify PMADs as a concern. There is no standardized documentation method, thus documentation style, the amount of detail, and the use of direct patient quotes remarkably varied.

## Results

### Demographics

Between November 2019 and December 2023, 14 patients endorsed having PMADs while being seen at AIMS, of which N = 3 (21.4%) met the inclusion criteria. Exclusion was primarily due to non-enrollment in the AMOS study. **Table 1** shows participants’ demographics and characteristics. Notably educational data was not available.

**Table 1:** Participant demographics and characteristics.

Characteristic	Participant 1	Participant 2	Participant 3
Race/ethnicity	Pacific Islander	White	White
Relationship status	Married	Married	Married
Occupation	Housekeeper	Disabled	Full-time employed
Age at treatment initiation	37 years	33 years	37 years
Months postpartum during treatment initiation with AIMS	8 months	20 months	24 months
Lactating during treatment	Yes	No	No
Breastfed during treatment	Yes	—	—
Number of children	2	1	2
Relevant obstetric history	C-section (1)	C-section (1)	Late-term pregnancy with induction (2); miscarriage (2)
Mental health condition arising in the perinatal period	No	Yes	Yes
Postpartum depression or anxiety	Depression	No	Anxiety

Participant 1 was actively breastfeeding during the initiation of KAP. She was notified to pump and save 12 hours’ worth of breast milk prior to KAP in order to replace the breastmilk that would be pumped and discarded in the 12 hours after KAP. No specific issues were noted related to lactation or breastfeeding while receiving 3 months of KAP. Participant 1 began the weaning process at 11 months post-partum due to depleting milk supply.

It is important to note that participant 3 received ketamine infusion therapy with another facility prior to receiving care at AIMS. She began ketamine therapy after her second miscarriage and paused during her last pregnancy. She also received two ketamine infusions within 12 months of childbirth. The specific postpartum timeframe when participant 3 initiated ketamine infusions was not specified.

### Appointments and treatments

**Table 2** shows participants’ mental health profiles and PMAD therapeutical approaches. When available, the specific psychotherapy modality (e.g., CBT, EMDR) was identified. Notably specific modalities were not always given, thus listed simply as psychotherapy. Their diagnosed depressive, anxiety, and trauma disorders are specified. No participant was documented to have bipolar disorder. Listed are concurrent medications and supplements specific to mental health or may impact mental health. During the course of therapy, participant 2 discontinued sertraline as it was ineffective and bupropion due to insomnia. Participant 3 was actively tapering off both prescription medications.

**Table 2:** Participant mental health profile and PMAD therapeutical approaches.

Variable	Participant 1	Participant 2	Participant 3
Depressive disorder	MDD, PPD	MDD	—
Anxiety disorder	ADU	GAD	ADU
Trauma disorder	PTSD	PTSD	—
Prescription medications	Sertraline 100 mg; bupropion XL 150 mg; fluoxetine 20 mg	Topiramate 100 mg; progesterone 100 mg; venlafaxine ER 225 mg	Lamotrigine 100 mg; sertraline 50 mg
PRN medications	Hydroxyzine 50 mg	Lorazepam 1 mg BID; Hydromorphone 2–4 mg hourly	—
OTC medications	Doxylamine 25 mg	—	—
Supplements	Prenatal vitamin; melatonin; DopaTone; botanical tincture (gotu kola, St. John’s wort, rosemary, bacopa, ginkgo biloba, ginger)	CBD oil	Vitamin D 3000 IU/day; omega-3–150 mg/day
Other therapeutic modalities	EMDR; PSYC	CBT; EMDR; PSYC	KET; PSYC
Number of appointments	18	29	7
Length of treatment (days)	168	323	54
Reason for discontinuation	Stipulated consent to review data only to a specific date	Cost prohibitive to continue	Ceased clinic engagement

ADU, anxiety disorder, unspecified; GAD, generalized anxiety disorder; MDD, major depressive disorder; PPD, postpartum depression; PTSD, post-traumatic stress disorder; CBT, cognitive behavioral therapy; EMDR, eye movement desensitization and reprocessing; KET, ketamine infusion; PSYC, psychotherapy, unspecified

The number of appointments are related to ketamine evaluations, experiential sessions, integration sessions, and general follow-up.

**Table 3** delineates each participant’s ketamine treatment information. All participants experienced side effects from ketamine that were mild and resolved within a day. Participants reported dizziness, mild nausea (with no reported episodes of emesis), sound sensitivity, and headache. One participant reported injection site tenderness; however, no further interventions were required. No side effects were noted for any infants fed by lactation.

**Table 3:** Participant ketamine treatment information.

Pt	In-clinic KAP				Home KAP				
	KS	Route	Dose	IS	KS	Route	Dose	Frequency	IS
1	4	IM	1.7 - 2.4 mg/kg	4	6	SL	200 mg - 400 mg	Every 2 weeks	4
2	12	IM	1.1 - 1.8 mg/kg	12	9	SL	250 mg - 300 mg	Weekly	4
3	1	SL	200 mg + 100 mg layering	1	—	—	—	—	—

KS, ketamine session; IS, integration session; IM, intramuscular; SL, sublingual.

### Data

#### Participant 1

*Ketamine Session:* The participant reported profound internal experiences characterized by feelings of safety, groundedness, and a sense of returning “home,” accompanied by newly acquired personal agency. A physical release of tension and pain was observed. Improvements in mental well-being were documented (“life feels pleasurable”), along with a perception of being “rooted and centered.” Deepening of familial connections were noted (“my daughter is a part of me;” “even when I don’t feel so good I still try to be present”) and anxieties concerning in-laws were processed, leading to a resolution to “delegate more.” The participant identified internal “resources to cope.”

*Integration Session:* The participant confirmed enhanced coping mechanisms, expressing an increased capacity for tolerance and a perception of life as “less overwhelming.” Hopefulness was articulated, and personal control was reasserted. Physical trauma release was reported. The participant initiated couples therapy and prioritized maintaining presence with children, acknowledging challenges related to self-care. The process of “reparenting self to be more gentle” and acquiring “more tools” was described. The clinician corroborated KAP’s efficacy for “depression/anxiety” and enhanced “coping.”

#### Participant 2

*Ketamine Session:* Brief notes indicated “discord with MIL” and “recent stressors.” KAP was deemed “helpful for mood” and the participant reported feeling “relaxed.” The initial KAP experience was likened to “Alice going down the rabbit hole.”

*Integration Session:* The participant did not notice any immediate benefits to her anxiety and depression symptoms after the first session. Experiences varied, with the participant feeling “on a rollercoaster” but noting “no changes in anxiety” after certain sessions. It was observed that “KAP is not unpleasant and does better on higher doses.” Mental health improvements included “improvement in insomnia,” “mood good,” and a more “relaxed” state. The participant had recurring disagreements with her mother regarding child-rearing. KAP was found to facilitate “emotional processing” post-session and complemented other therapeutic modalities such as individual therapy and EMDR.

#### Participant 3

*Ketamine Session:* The participant experienced strong positive affirmations (“good mom, strong, confident beautiful, I love myself”) and “big love for daughter husband and self.” Visuals, including “colors and shapes” and a “little girl,” were noted, along with a sensation of “coming home to myself.” Inherent positive qualities were recognized.

*Integration Session:* After her ketamine session, the participant felt “more present and focused, pace of thinking has slowed, listening better.” Ketamine visuals were revisited for reflection, and the participant aimed to address internal challenges (“doesn’t want to be afraid of her darkness”) and goals (“‘I hope I am building a relationship with my girls like I have with my mom”). “Fierceness” and “coming home to self” were reinforced. The initial “big love” was translated into goals for building “close relationship with daughters” and envisioning an improved life. The family observed a reduction in anxiety, which benefited the entire family system. The participant shared “hopefully [ketamine] helps more postpartum women; it actually does something in your brain that can wake up parts that have been clamped down because of stress.”

## Discussion

Ketamine-assisted psychotherapy (KAP) as an adjunct therapy to traditional psychiatric treatments positively impacted all three participants with PMADs, improving mental well-being, coping, and relationships. Participant 1 experienced profound shifts to perspective, increased agency, and reduced tension. Participant 2 noted improvements to mood and insomnia and better emotional processing. Participant 3 reported strong self-affirmations and reduced anxiety. Integration sessions reinforced these gains, helping with coping, self-care, and family dynamics. KAP was correlated with improvements to anxiety and unipolar depression, and enhancing coping mechanisms.

Overall, ketamine sessions primarily facilitated profound internal experiences, emotional release, and the initial emergence of self-awareness and agency. These were often marked by vivid imagery and deep introspection. In contrast, integration sessions served as a crucial space for processing these insights, translating them into practical coping strategies, addressing existing family dynamics, and reinforcing the sustained application of newfound resilience and self-care in daily life.

Research suggests high stress and history of prenatal psychiatric disorders can be a strong risk factor for developing PMADs (12) and study participants demonstrated these expected risk factors as well. All three participants had a history of prenatal psychiatric conditions and some type of anxiety disorder. Two of the three participants had mental health diagnoses precipitate during pregnancy. Two of three participants had diagnoses for depression and PTSD. This suggests that the therapeutic benefits of ketamine may, in part, stem from its ability to address multiple psychopathologies (32, 39), including those rooted in trauma. If underlying risk factors like trauma, which are highly correlated with the development of PMADs (12, 13), are concurrently mitigated by ketamine treatment, it presents a compelling argument to consider ketamine’s broader application.

Although participants were all receiving concurrent psychiatric treatments, their desire to seek KAP as a complementary treatment may indicate efficacy in more treatment-resistant PMADs. However, it is crucial to acknowledge that this sample may be disproportionate, consisting of individuals who sought ketamine after other conventional treatments had not provided sufficient relief. This self-selection bias warrants further investigation in larger, more diverse cohorts.

This study also underscored the importance of intentional administration and integrated therapy. As all participants received ketamine as part of a protocol, this supports that the therapeutic effects of ketamine are enhanced when coupled with psychological support (30, 47, 55), allowing individuals to process their experiences and integrate insights gained during treatment. This finding highlights the need for a holistic approach to ketamine-assisted psychotherapy in PMADs.

The tolerability profile of ketamine treatment in this study was favorable. Participants reported only mild side effects, which typically resolved within a day, consistent with general findings among those who have received ketamine within the population (47, 49). Crucially, no side effects were reported for infants, which is a vital consideration when treating individuals in the perinatal period. This benign safety profile further strengthens the argument for ketamine as a viable treatment adjunct for PMADs, particularly given the vulnerability of this population.

In summary, this study provides a precedent for the benefits of ketamine treatment in PMADs. Integration sessions following ketamine-assisted psychotherapy consistently demonstrated significant, overarching benefits for participants’ family systems. These benefits stemmed from reinforced personal insights and the development of practical coping strategies that transcended individual experiences. Participants exhibited a newfound sense of agency, leading to proactive engagement in improving family dynamics, such as initiating couples therapy or actively maintaining presence with children. The therapeutic space provided by integration sessions facilitated crucial emotional processing of familial discord. Profound internal shifts experienced during KAP, when effectively integrated, translated into tangible improvements in family harmony, reduced anxiety within the household, and a clearer vision for desired family relationships. The observed efficacy, coupled with its fair tolerability and the absence of infant side effects, suggests that ketamine has the potential to revolutionize the treatment landscape for these debilitating conditions. Future research should focus on larger-scale, randomized controlled trials to further validate these findings and explore optimal dosing regimens and long-term outcomes.

### Limitations

This study’s small sample size may not represent all PMAD individuals. As insurance typically does not cover ketamine for mood disorders (except patented IN esketamine), direct costs for an in-clinic IM or SL session range from $500-$700 (58). The likelihood of participants affording ketamine suggests a sample skewed towards higher socioeconomic status (SES). Lower SES individuals, more prone to PMADs, may benefit from ketamine but lack access due to cost.

As a retrospective study, obtaining complete family system information was challenging. Other PMAD risk factors (e.g., lack of spousal support, epigenetics) were unknown. All participants in this study have heterosexual gender orientation. Findings from this study cannot provide any correlations for non-heterosexual co-parents.

Clinician documentation varied, from detailed notes to concise summaries. This lack of standardized documentation may have resulted in lost patient narratives.

## Conclusion

Ketamine therapy, including KAP, is emerging as a promising treatment option for a variety of psychiatric disorders (47). This data represented individuals who received ketamine therapy while having PMADs within 24 months of childbirth and experienced general improvement in mental health symptoms, increased psychosocial self-awareness, and improvement of feelings of self-worth and self-efficacy. PMADs represent a severe and concerning form of psychiatric illness. ketamine as an adjunctive therapy enhances mental health outcomes compared to standalone, current FDA approved treatments. However, there is a limited number of high quality randomized controlled trials to better understand the efficacy of this treatment and durability of its effects in various conditions. To further understand ketamine’s role in perinatal mental health, randomized control trials of ketamine for PMAD prophylaxis should be compared to ketamine treatments for existing PMADs. In the interim, retrospective case series such as this can help fill this gap in the literature and provide a precedent for ketamine treatment in PMADs.

## Data Availability

The raw data supporting the conclusions of this article will be made available by the authors, without undue reservation.
